# Multiparametric MRI Assessment of Morpho-Functional Muscle Changes Following a 6-Month FES-Cycling Training Program: Pilot Study in People With a Complete Spinal Cord Injury

**DOI:** 10.2196/64825

**Published:** 2025-01-16

**Authors:** Alfonso Mastropietro, Denis Peruzzo, Maria Giovanna Taccogna, Nicole Sanna, Nicola Casali, Roberta Nossa, Emilia Biffi, Emilia Ambrosini, Alessandra Pedrocchi, Giovanna Rizzo

**Affiliations:** 1 Istituto di Sistemi e Tecnologie Industriali Intelligenti per il Manifatturiero Avanzato, Consiglio Nazionale delle Ricerche, via Alfonso Corti, 12, Milan, 20133, Italy, 39 02 2369 993; 2 Neuroimaging Unit, Scientific Institute, IRCCS E. Medea, Bosisio Parini, Lecco, Italy; 3 Istituto di Tecnologie Biomediche, Consiglio Nazionale delle Ricerche, Segrate, Italy; 4 Dipartimento di Ingegneria Meccanica, Politecnico di Milano, Milan, Italy; 5 WeCobot Lab, Polo Territoriale di Lecco, Politecnico di Milano, Lecco, Italy; 6 Dipartimento di Elettronica, Informatica e Bioingegneria, Politecnico di Milano, Milan, Italy; 7 Laboratorio di Bioingegneria, Istituto di Ricovero e Cura a Carattere Scientifico Eugenio Medea, Bosisio Parini, Italy; 8 Bioengineering Lab, Scientific Institute, IRCCS E. Medea, Bosisio Parini, Lecco, Italy; 9 Nearlab, Dipartimento di Elettronica, Informatica e Bioingegneria, Politecnico di Milano, Milan, Italy

**Keywords:** functional electrical stimulation, FES, cycling, exercise, physical activity, spinal cord injury, multiparametric MRI, skeletal muscle, rehabilitation, magnetic resonance imaging, muscle, muscular, musculoskeletal, spine, MRI, mpMRI, image, imaging

## Abstract

**Background:**

Spinal cord injuries (SCIs) cause debilitating secondary conditions such as severe muscle deterioration, cardiovascular, and metabolic dysfunctions, significantly impacting patients’ quality of life. Functional electrical stimulation (FES) combined with cycling exercise (FES-cycling) has shown promise in improving muscle function and health in individuals with SCI.

**Objective:**

This pilot study aimed to investigate the potential role of multiparametric magnetic resonance imaging (MRI) to assess muscle health during and after an FES-cycling rehabilitation program.

**Methods:**

Four male participants with chronic SCI underwent a 6-month FES-cycling training program, consisting of two 30-minute sessions per week. MRI scans were performed at baseline (T_0_), after 3 months (T_1_), at the end of the training (T_2_), and 1-month posttraining (T_3_). The MRI protocol included T_1_-weighted imaging for volume quantification, Dixon imaging for fat fraction, multi-echo spin echo for T_2_ relaxation times, and diffusion tensor imaging to assess diffusion parameters.

**Results:**

Muscle hypertrophy was observed, with an average increase in muscle volume of 22.3% at T_1_ and 36.7% at T_2_ compared with baseline. One month posttraining, muscle volume remained 23.2% higher than baseline. Fat fraction decreased from 11.1% at T_0_ to 9.1% at T_2_, with a rebound to 10.9% at T_3_. T_2_ relaxation times showed a reduction even though this was not consistent among participants. Diffusion tensor imaging parameters revealed subtle changes in muscle tissue microstructure, with a decrease in fractional anisotropy mainly associated to an increase of radial diffusivity.

**Conclusions:**

Although preliminary, this study provides evidence that 6 months of low-intensity FES-bike training can increase muscle volume and decrease fat infiltration in individuals with SCI. The study demonstrates that the use of a multiparametric MRI provides comprehensive insights into both macroscopic and microscopic changes within muscle tissues, supporting its integration into clinical practice for assessing the efficacy of rehabilitation interventions.

## Introduction

Spinal cord injury (SCI) refers to a damage of the spinal cord due to traumatic or nontraumatic events affecting globally over 15 million people [1]. SCIs cause debilitating and life threating secondary conditions that leads to critical health complications, such as severe muscle deterioration, weakness, cardiovascular, and metabolic dysfunctions [1-3], significantly impacting patients’ quality of life. Due to the unloading following an SCI, skeletal muscle (SM) undergoes numerous adaptations, including rapid and profound atrophy, intramuscular fat accumulation, impaired muscular glucose metabolism and decreased force generation and muscle performance [4].

Therefore, to counteract detrimental effects of SCI on SM health, rehabilitation plays a crucial role with promising positive effects [4-7]. So far, several activity-based interventions have been widely applied in SCI and among them transcranial magnetic stimulation, functional electrical stimulation (FES), and robotic-assisted treadmill training are effective in improving function in individuals with SCI [5].

FES consists in the application of low-energy electrical stimuli to peripheral nerves, to promote muscle contractions which ultimately results in functional movements [8]. Specifically, FES-cycling training, which exploits the use of FES to induce the pedaling movement has shown promising results in enhancing muscle health and function in individuals with SCI [4,9] Previous studies demonstrated improvements in muscle mass, strength, and overall metabolic profile following FES-based training. The effects of FES-cycling training on muscle health after SCI are multifaceted and include muscle atrophy attenuation or reversal [10-12], increased muscle cross-sectional area (CSA) [13-15], increased muscle size [10], improved body composition, plasma glucose, and SM glucose uptake [16-18], increased power output, peak isometric strength, knee extensor torque [13,19,20], and increased motor function scores [19].

Currently, in this field, traditional noninvasive assessment methods for SM health fall short in providing comprehensive insights into muscle morphology and function. In particular, the use of MRI (magnetic resonance imaging) is limited to basic protocols, typically consisting of T_1_-weighted sequences, primarily focused on the quantification of muscle volume and CSA [13,15,19,21-24] thus not fully exploiting the strength and versatility of noninvasive MRI techniques in capturing other crucial aspects such as fat infiltration, tissue inflammation, and microstructural changes.

Multiparametric MRI (mpMRI) addresses these limitations by integrating various imaging sequences, including T_1_-weighted (T_1_w), quantitative T_2_ (qT_2_), diffusion-weighted imaging (DWI), and Dixon techniques [25,26], offering a detailed quantitative evaluation of muscle properties and enabling a thorough assessment of muscle health.

MpMRI provides several advantages over traditional MRI to evaluate SM. It allows for the quantification of fat and water content within muscles, which is crucial for understanding the extent of fat infiltration, a common issue in individuals with SCI. In addition, parameters such as T_2_ relaxation times and diffusion related parameters provide information on muscle edema, inflammation, and microstructural properties, essential for a comprehensive assessment of SM tissue [27]. This more advanced approach thus holds significant potential for monitoring the SM changes due to rehabilitation interventions like FES-cycling training.

This pilot study aims to leverage the capabilities of mpMRI to assess the morphological and functional changes and their evolution in SM of individuals with complete SCI following FES-cycling training. This study is part of a wider one evaluating the effects of FES-cycling training on multi-factorial aspects, such as osteoporosis, pedaling performance, spasticity and perceived well-being of patients [28].

By providing a detailed evaluation of muscle health, this study seeks to explore the feasibility of the use of mpMRI to enhance our understanding of the impact of FES-cycling training on muscle tissue and promote its implementation as a valuable tool to assess rehabilitation effects on SM in individuals with complete SCI.

## Methods

### Participants

A total of 4 male participants with complete SCI, aged 30 (SD 8) years, were recruited from the Istituto di Ricovero e Cura a Carattere Scientifico Eugenio Medea. The inclusion criteria required participants to have a complete SCI (more than 6 month and less than 5 years after the lesion; American Spinal Injury Association Impairment Scale of A or B; lesion level ≤ T_3_), an age between 18 and 65 years old, and the ability to engage in the FES-cycling training program.

At baseline (T_0_) demographic and clinical data were collected from each participant, including age, height, BMI, time since injury, lesion level (American Spinal Injury Association Impairment Scale), previous experience with FES and trike or cycling after the injury, current pharmacological therapy. Furthermore, spasticity was assessed using the Modified Ashworth Scale (MAS) and no severe levels were found at T_0_ for all participants (MAS score for each participant <2).

### Functional Electrical Stimulation–Cycling Training Program

The participants underwent a 6-month FES-cycling training program performed using a recumbent trike (ICE VTX, 2017), adapted for participants with reduced mobility, combined with two 4-channels current-controlled stimulators (RehaMove3 and Hasomed GmbH) [29]. The training program included 2 weekly sessions, each lasting 30 minutes of stimulation, over 6 months. Each session consisted of 6 sets of 3‐6 minutes of duration at a cycling rate between 30 and 50 revolutions per minute, with brief rest periods in between. The cadence for each pilot was chosen to allow independent pedaling throughout the set duration [28]. Throughout the training sessions, continuous monitoring of heart rate (HR) was conducted with the only purpose of ensuring participant safety, allowing for the immediate cessation of exercise if HR exceeded safe thresholds. This was achieved using the Polar H10 chest strap, which captures HR data at a sampling rate of 1 Hz. HR during training sessions ranged from a minimum average HR of 75 bpm to a maximum average HR of 113 bpm.

The stimulators delivered biphasic square pulses with a maximum current amplitude of 130 mA, a frequency of 40 Hz and a pulse width that ranged between 400 and 500 µs. The stimulation targeted 4 muscle groups per leg such as quadriceps, hamstrings, gluteal muscles, and calf muscles.

### Magnetic Resonance Imaging Acquisitions

MRI scans were performed at 4 time points, at the beginning of the study (T_0_, n=4), after 3 months of training (T_1_, n=4), at the end of the training (T_2_, n=4), and 1-month posttraining deconditioning (T_3_, n=3). One of the 4 participants was scanned only up to T_2_, since he did not interrupt the training program. The longitudinal MRI assessment was designed to track the SM alterations that occur throughout the training period. In addition, this timeline allows us to assess the impact of short-term deconditioning, which can frequently arise in real-life situations, such as during vacation intervals.

A 3T Achieva dStream MRI scanner (Philips) was used for imaging. The participant was positioned on the examination table, feet facing the scanner (“feet-first” orientation), with the pelvis slightly shifted to align the thigh being scanned closer to the midline. Positioning cushions were used to improve comfort, stabilize the limb and keep the legs separated. Finally, the multichannel Philips dStream Torso coil is placed over the targeted thigh and secured with velcro straps.

**Figure 1.**
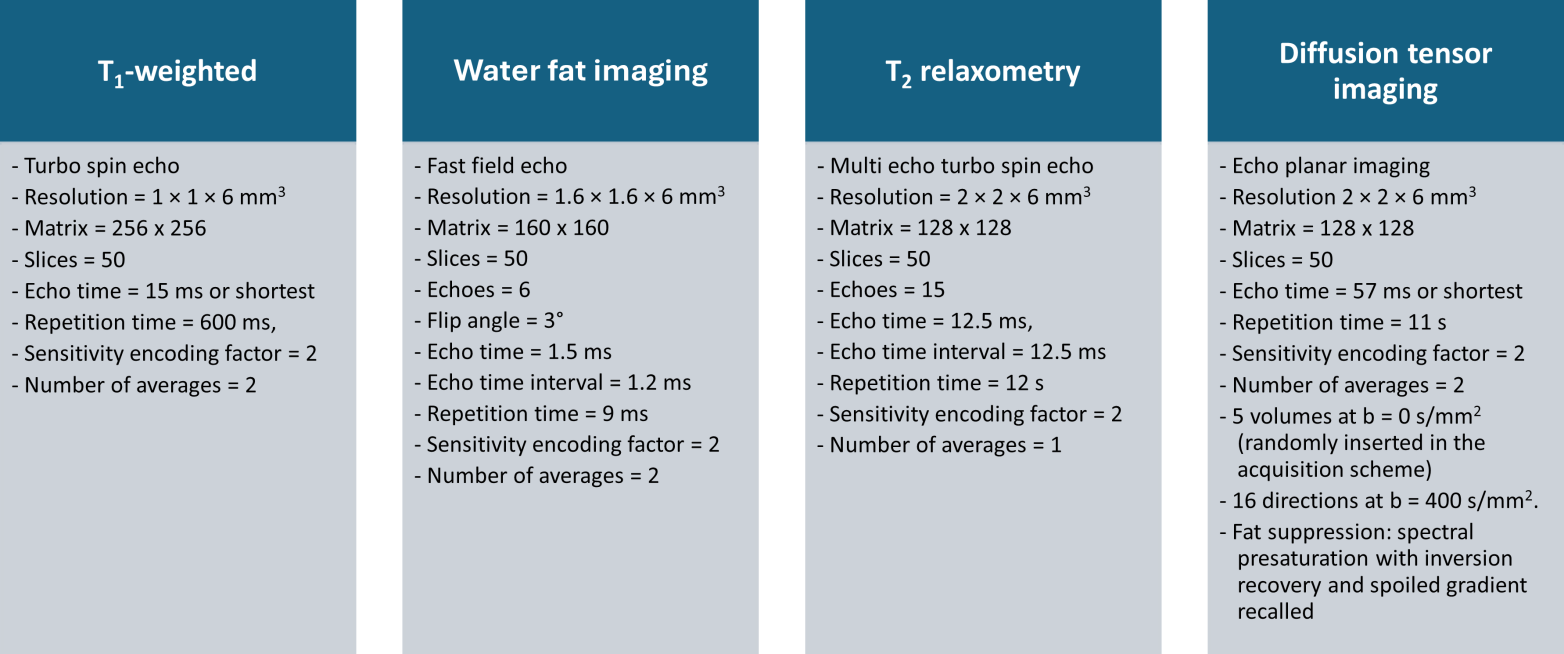
Multiparametric magnetic resonance imaging protocol used for assessing muscle volume, fat fraction, T_2_ relaxation times, and diffusion parameters. All sequences were acquired according to the axial plane placed perpendicular to the femur and with the upper part of the field of view placed in the middle of the head of the femur. All sequences share the same field of view (size 256 × 256 × 300 mm^3^), regardless of their acquisition matrix and reconstructed voxel size.

The MRI protocol included a T_1_w turbo spin echo (TSE) sequence for volume quantification, a 6-point Fast Field Echo m-Dixon Quant sequence for fat fraction quantification, 15-echo multi-echo turbo spin echo (multi-TSE) sequence for T_2_ relaxation time quantification, and a single shell diffusion tensor imaging (DTI; 16 directions at b=400 s/mm²; 5 b=0 s/mm^2^ volumes acquired also in opposite phase encoding direction) for the diffusion parameters assessment. Regarding the anatomical region covered in the magnetic resonance (MR) images, the scans encompassed thigh volume in a 30 cm range along the head-to-feet axis, starting from the midpoint of the femoral head. Further details regarding the acquisition protocols are displayed in Figure 1.

### Magnetic Resonance Imaging Analysis

**Figure 2.**
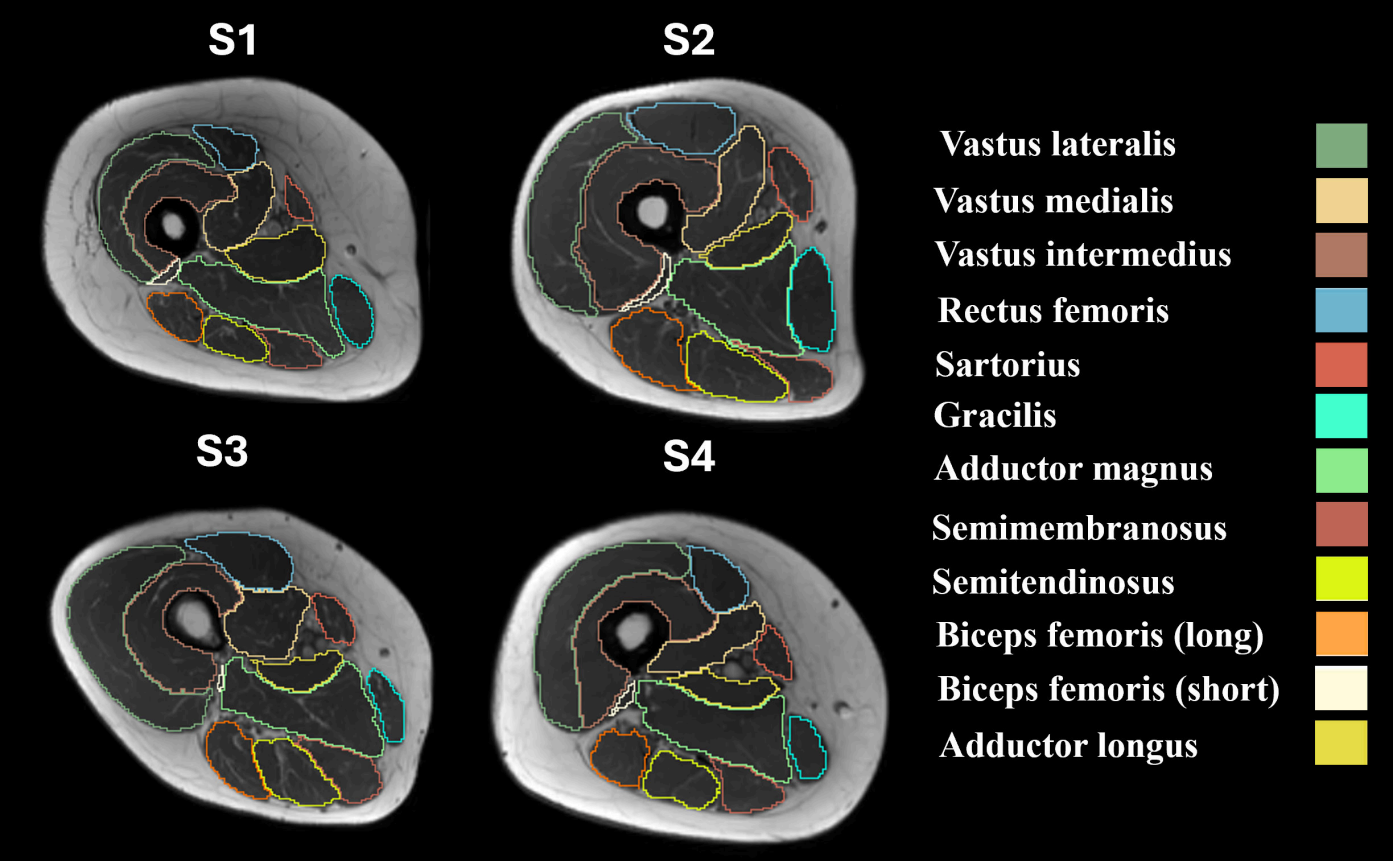
Representative axial magnetic resonance imaging cross-sections of the thigh for 4 participants (S1 to S4), with segmented anatomical regions representing individual muscles. Each muscle is outlined in a specific color corresponding to the legend on the right. Muscles include the vastus lateralis, vastus medialis, vastus intermedius, rectus femoris, sartorius, gracilis, adductor magnus, semimembranosus, semitendinosus, biceps femoris (long and short heads), and adductor longus.

As shown in Figure 2, to derive muscle volume and CSA, regions of interest (ROIs) were delineated on T_1_w images for specific thigh muscles, including the vastus lareralis, vastus medialis, vastus intermedius, rectus femoris, sartorius, gracilis, adductor magnus, semimembranosus, semitendinosus, biceps femoris caput longum, biceps femoris caput breve, and adductor longus. The semiautomated segmentation was performed using the Deep Anatomical Federated Network [30], which combines automated and manual refinement processes. Volumes for individual muscles and the overall average were computed using 3DSlicer software. The largest CSA (CSA-max) was obtained selecting the slice exhibiting the maximum muscle area.

**Figure 3.**
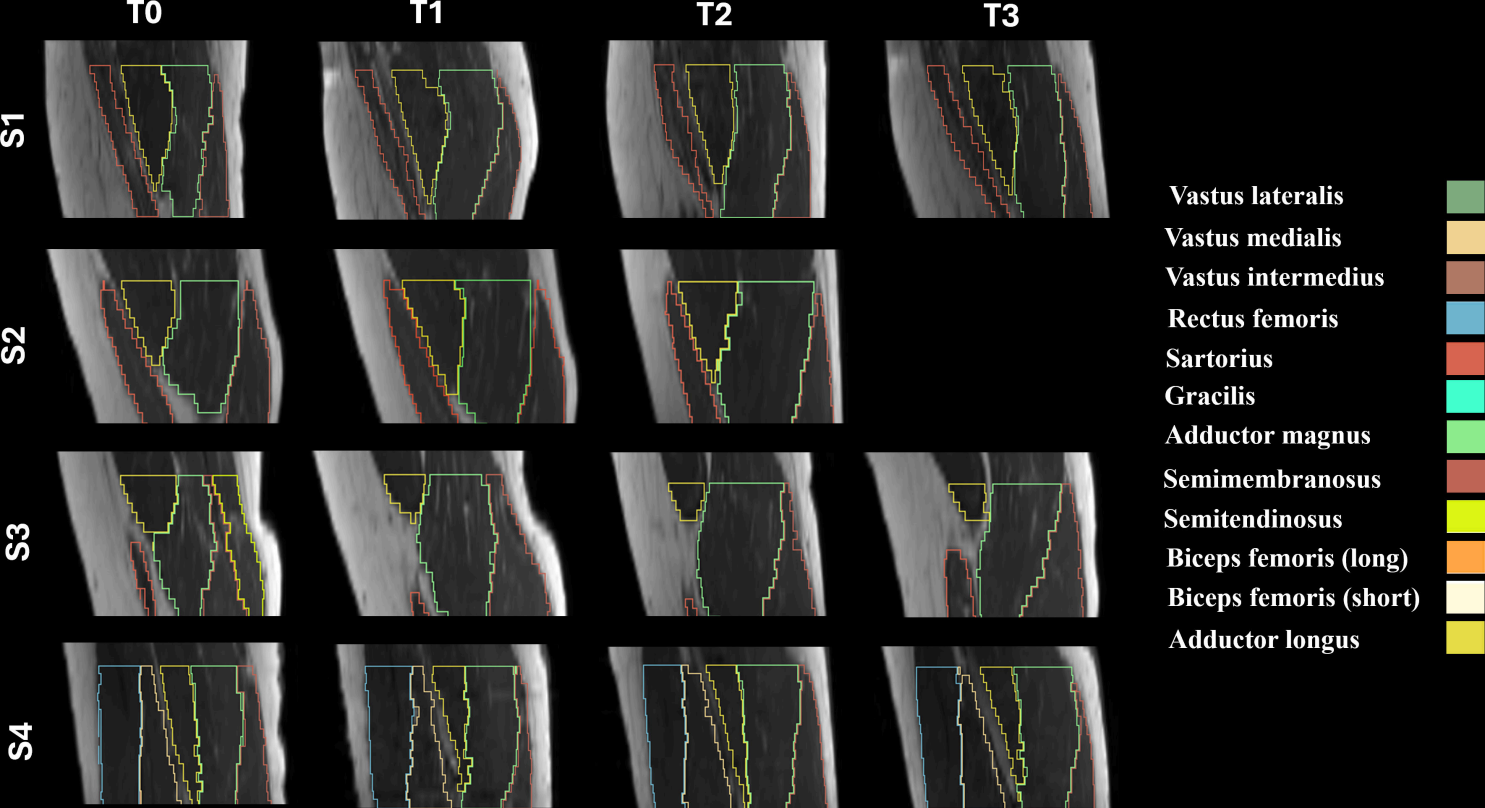
Extension of thigh muscle regions of interest across 4 participants (S1‐S4) in a representative sagittal plane for each time point (**T_0_-T_3_**). Each muscle is outlined with a distinct color. The regions of interest boundaries were consistently defined from the beginning of the semimembranosus to the last available slice of the rectus femoris, ensuring reproducibility across participants and time points. Magnetic resonance imaging scans covered a 30 cm range along the head-to-feet axis from the midpoint of the femoral head.

For the quantitative analysis of MRI derived parameters, the same ROIs used for the evaluation of volume and CSA, ranging from the beginning of the semimembranosus to the last available slice of the rectus femoris were used. Consistency was maintained in the ROIs across all longitudinal scans for each participant as shown in Figure 3.

The parameters derived from the MR images were calculated by averaging their values across the 12 distinct ROIs defined before. The corresponding coefficients of variation were also calculated to characterize the intrinsic variability of each parameter for each time point and each participant (Multimedia Appendix 1).

Fat fraction (FF) was estimated from the mDixon sequence [31] using the Quant model implemented in the scanner which improves the classical bicompartmental exponential model by including a 7-peak fat modeling and T_2_* correction to produce FF maps.

T_2_ relaxation times were estimated from the multi-TSE images using the extended phase graph approach [32], which models spin behavior and predicts MR signals at various time points, accounting for T_1_ and T_2_ relaxation processes. Specifically, an open-source toolkit for water T_2_ mapping was used [33]. The algorithm was applied to the multi-TSE acquisition after generating a dictionary containing 200 linearly spaced values for water T_2_ (range 5‐80 ms), 50 values for the B1 factor (range 50%‐120%), and 101 values for the FF (range 0%‐100%). The fat T_2_ was assumed constant at 151 ms. Maps derived from the extended phase graph method, constrained by the external proton-density-weighted fat fraction, were produced.

DTI parameters, including fractional anisotropy (FA), mean diffusivity (MD), radial diffusivity (RD), and axial diffusivity (AD), were calculated using the MRtrix3 package [34]. Images were denoised with a method based on random matrix theory [35] and the Gibbs ringing artefacts were removed using the method of local subvoxel-shifts [36]. To mitigate susceptibility artifacts, b_0_ images were collected with the reversed phase-encode directions, resulting in pairs of images with distortions going in opposite directions. From these pairs the susceptibility-induced off-resonance field was estimated using a method similar to that described in [37] as implemented in FMRIB Software Library [38]. Afterwards, images were corrected for eddy current-induced distortions and participant movements registering each volume in the data set to the reference b_0_ volume. Finally, the diffusion tensor was fitted to the log-signal using an iterative weighted least-squares with weights based on the empirical signal intensities (2 iterations were be performed) [39].

### Statistical Analysis

Considering the small sample size, a descriptive statistical analysis was performed using R Statistical Software (v4.1.2, R Foundation for Statistical Computing) [40,40]. In particular, changes in muscle volume, CSA, FF, T_2_ relaxation times, and DTI parameters across the 4 time points were described reporting medians and median absolute deviations (MAD).

### Ethical Considerations

All participants provided written informed consent before enrollment. The research protocol was approved by the ethics committee of Istituto di Ricovero e Cura a Carattere Scientifico Medea (N. 14/22 CE, approved on February 17, 2022) and the protocol was registered on ClinicalTrials.gov (NCT06321172).

## Results

### Participants

Table 1 provides clinical and demographic characteristics of participants in the pilot study. All participants completed the training program with a compliance greater than 78%, performing a minimum of 40 sessions over the 52 foreseen.

**Table 1.** Clinical and demographic characteristics of participants.

Participant	Age (years)	Distance from lesion (years)	Type of lesion (ASIA^a^)	Height (cm)	BMI at T_0_	Previous experience with FES^b^	Previous experience with cycling or trike	Drug therapy
S1	23	2.0	T10-11 (A)	178	25.2	No	No	—^c^
S2	29	1.1	T3 (A)	173	20.7	Yes	No	Oxybutyn, lansoprazole, D-base
S3	41	3.8	T5 (A)	175	24.5	Yes	Yes	—
S4	27	1.8	T12 (B)	191	21.9	Yes	Yes	Baclofen, lyrica

a ASIA: American Spinal Injury Association Impairment Scale.

b FES: functional electrical stimulation.

c Not applicable.

### Muscle Volume and Cross-Sectional Area

**Figure 4.**
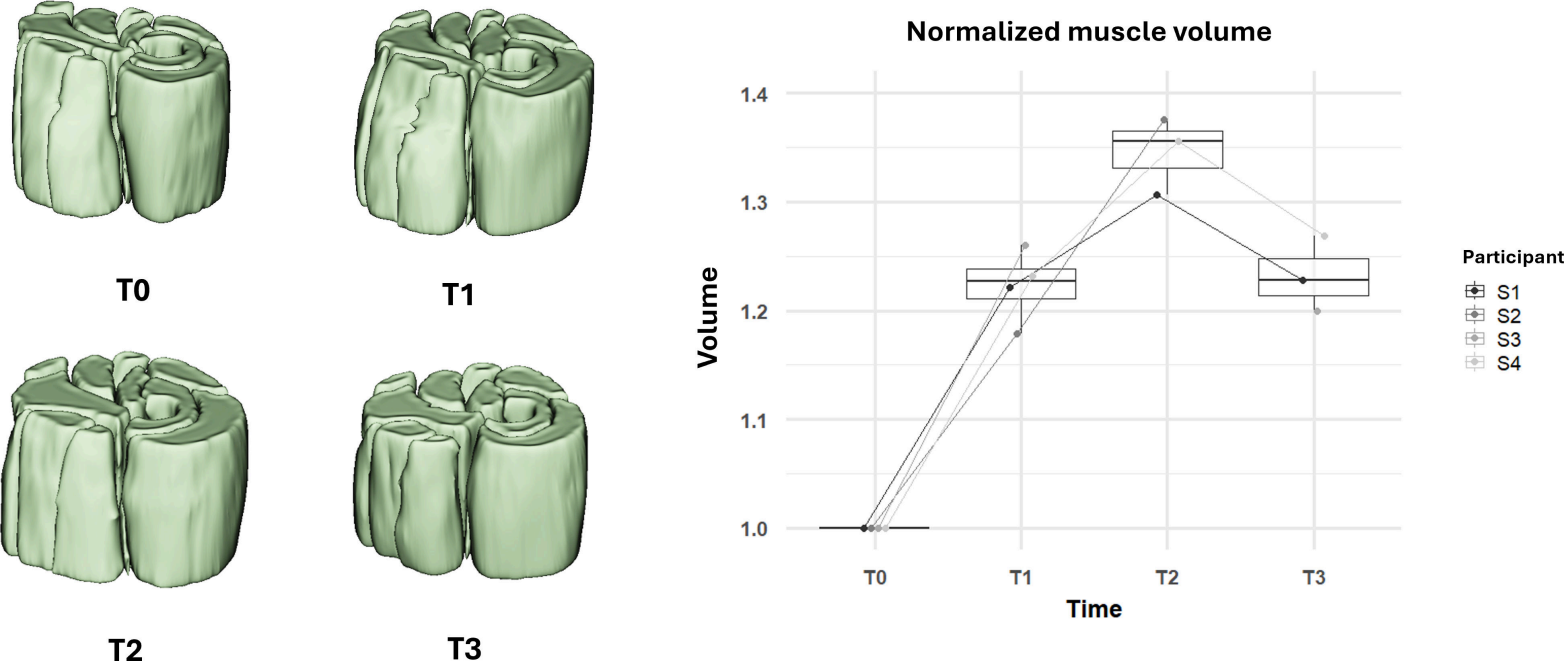
Changes in normalized muscle volume over the 6-month functional electrical stimulation–cycling training and 1-month posttraining. The left panel shows 3D-reconstructed volume renderings for an example volunteer at each time point (**T_0_, T_1_, T_2_, and T_3_**), illustrating the changes in muscle size. The right panel presents box plots of normalized muscle volume measurements for all participants at each time point.

**Figure 5.**
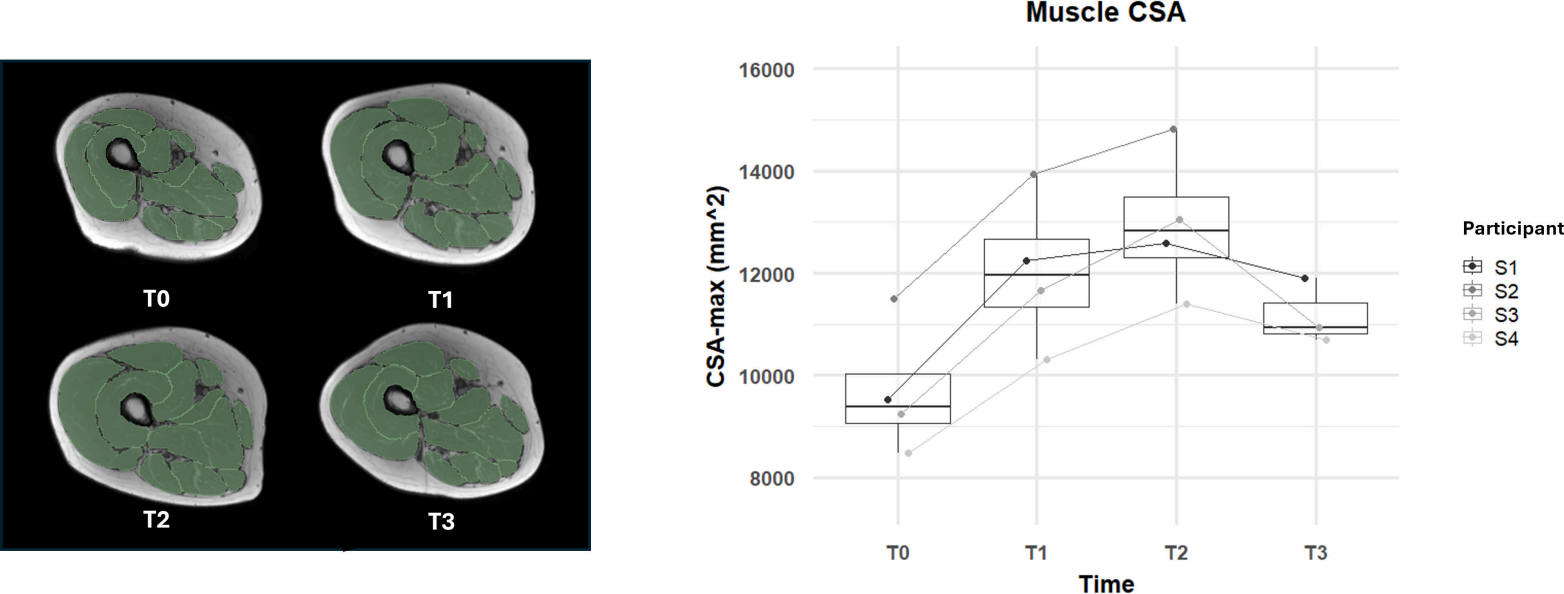
Changes in a muscle’s largest cross-sectional area (CSA) over the 6-month functional electrical stimulation–cycling training program and 1-month posttraining. The right panel shows box plots of cross-sectional area measurements for all participants at each time point (**T_0_, T_1_, T_2_, and T_3_**). The left panel illustrates the magnetic resonance imaging cross-sectional images of the thigh muscles for an example volunteer at each time point, highlighting the visual changes in muscle size.

As shown in Figure 4, analysis of the MR images revealed relevant changes in muscle volume and CSA-max over the 24-week FES-bike training program. At T_1_ (after 3 months of training), participants showed an average increase in muscle volume of 23% compared with baseline (T_0_). This growth continued through T_2_ (after 6 months of training), with an overall increase of 37% in muscle volume. One month after the training (T_3_), a slight reduction was observed, yet the muscle volume remained 23% higher than at baseline. Similarly, as displayed in Figure 5, CSA-max increased by 27% at T_1_ and 37% at T_2_, with a decrease to 16% above baseline at T_3_. It is noteworthy that the observed trend was consistent in all the 4 participants involved in this study as shown in Figures3  4.

### Fat Fraction

**Figure 6.**
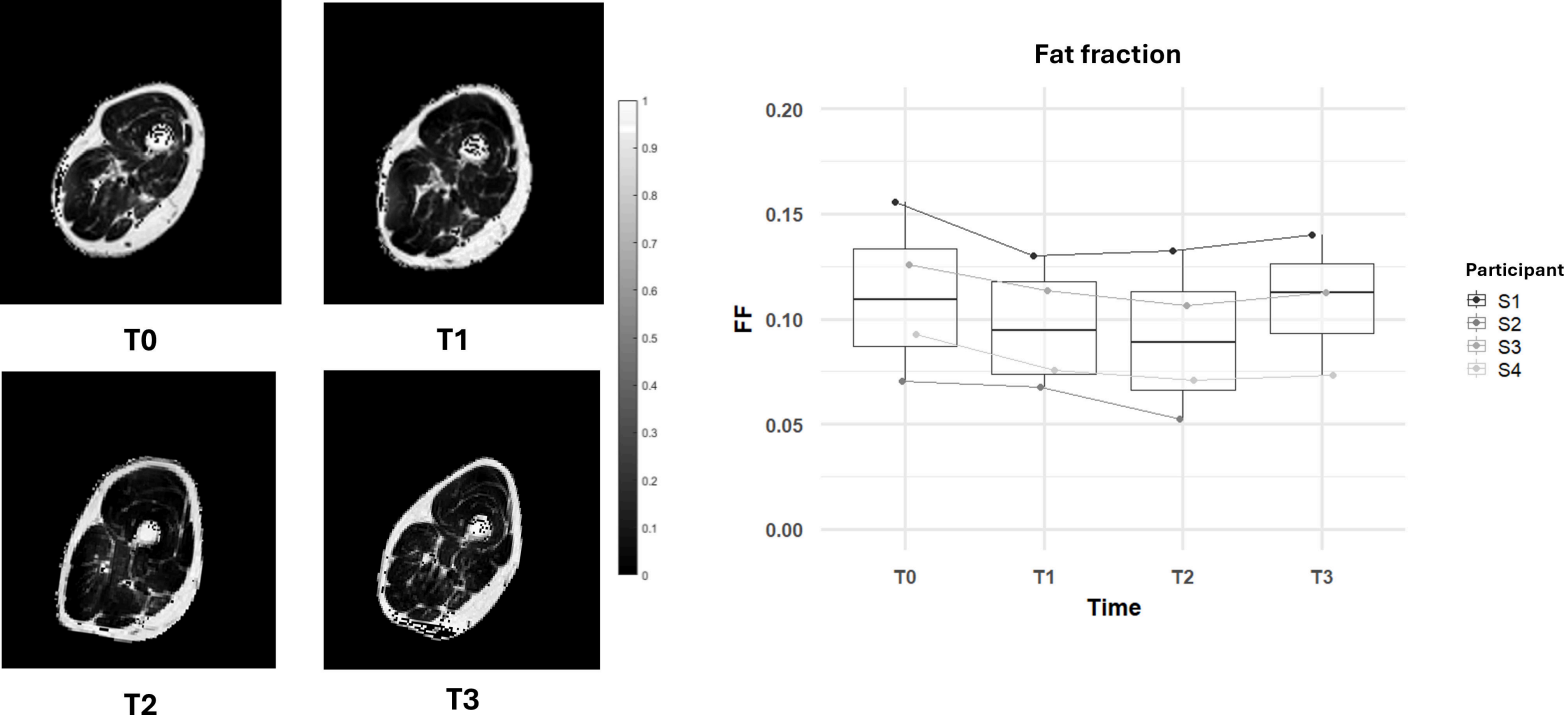
Changes in muscle fat fraction over the 6-month functional electrical stimulation–cycling training and 1-month posttraining. The right panel shows box plots of fat fraction measurements for all participants at each time point (**T_0_, T_1_, T_2_, and T_3_**). The left panel displays magnetic resonance imaging cross-sectional images of the thigh muscles for an example volunteer at each time point, illustrating the reduction in fat infiltration.

As shown in Figure 6, the FF in the muscle tissue showed a notable decrease from T_0_ to T_2_. The baseline measurements indicated a median FF of 10.9% (MAD=4%). By T_1_, the FF decreased to 9.4% (MAD=3%), representing a reduction of 13%. By T_2_, this reduction continued to 8.9% (MAD=4%), representing a total reduction of approximately 19%. The FF reduction was consistent in all the 4 participants.

At T_3_, a rebound effect was observed, with FF measurements showing an increase to 11.3% (MAD=4%), a 3% higher than the baseline. Even in this case the trend was consistent in all the participants.

### T_2_ Relaxation Time

**Figure 7.**
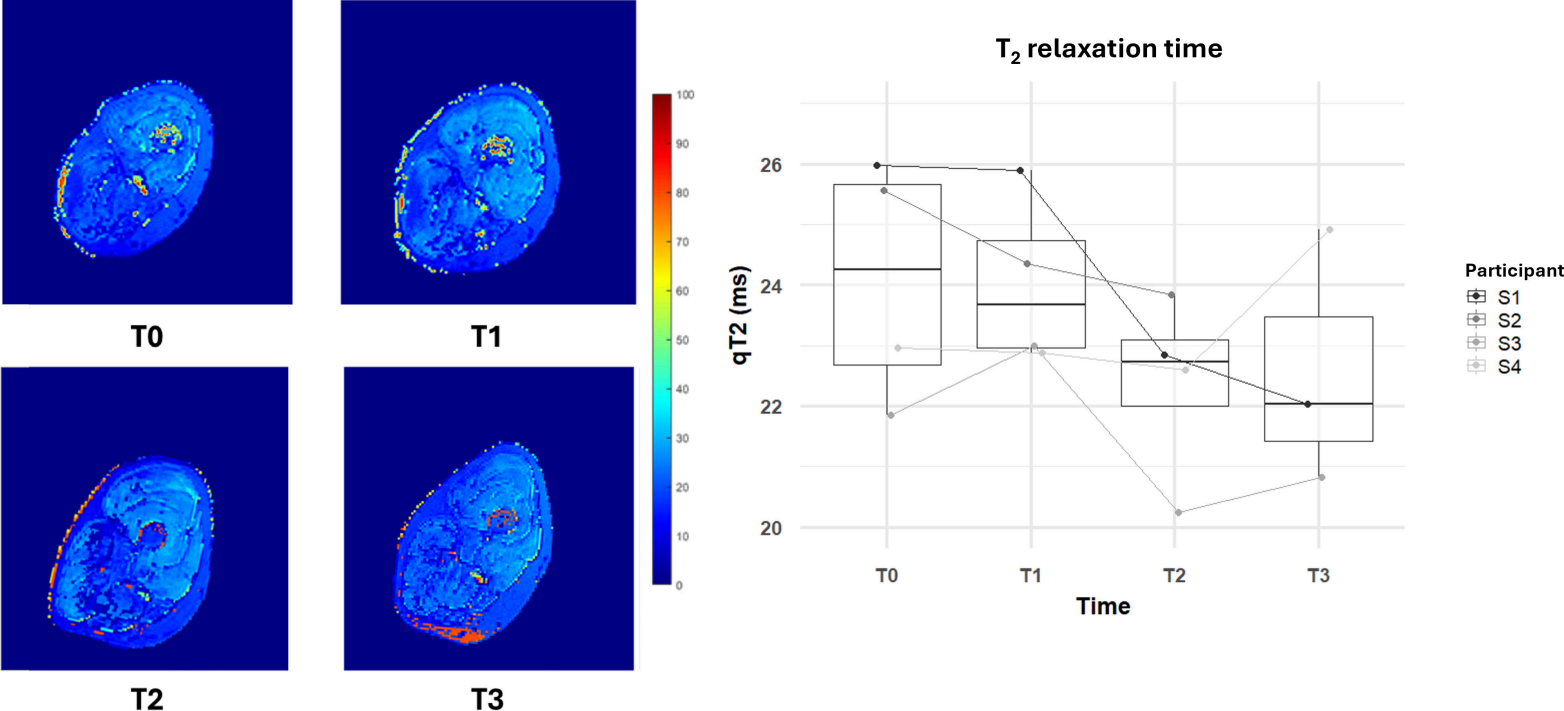
Changes in T_2_ relaxation times over the 6-month functional electrical stimulation–cycling training and 1-month posttraining. The right panel shows box plots of T_2_ relaxation times for all participants at each time point (**T_0_, T_1_, T_2_, and T_3_**). The left panel displays T_2_ relaxation maps of the thigh muscles for an example volunteer at each time point.

As shown in Figure 7, qT_2_ times demonstrated a decreasing trend over the course of the training. Starting at a median of 24.3 ms (MAD=2.2 ms) at T_0_, the qT_2_ reduced to 23.7 ms (MAD=1.1 ms) by T_1_, reflecting a decrease of 2%. By T_2_, the qT_2_ relaxation time further reduced to 22.7 ms (MAD=0.9 ms), reflecting a total decrease of approximately 6%. At T_3_, the qT_2_ relaxation time slightly reduced to 22.0 ms (MAD=1.8 ms) and it was still lower than the initial value of about 9%. As to qT_2,_ there was a high variability, and the trend was not consistent among participants. Just at T_2_, 4 out of 4 participants showed a reduction in qT_2_ as shown in Figure 7.

### Diffusion Tensor Imaging Parameters

**Figure 8.**
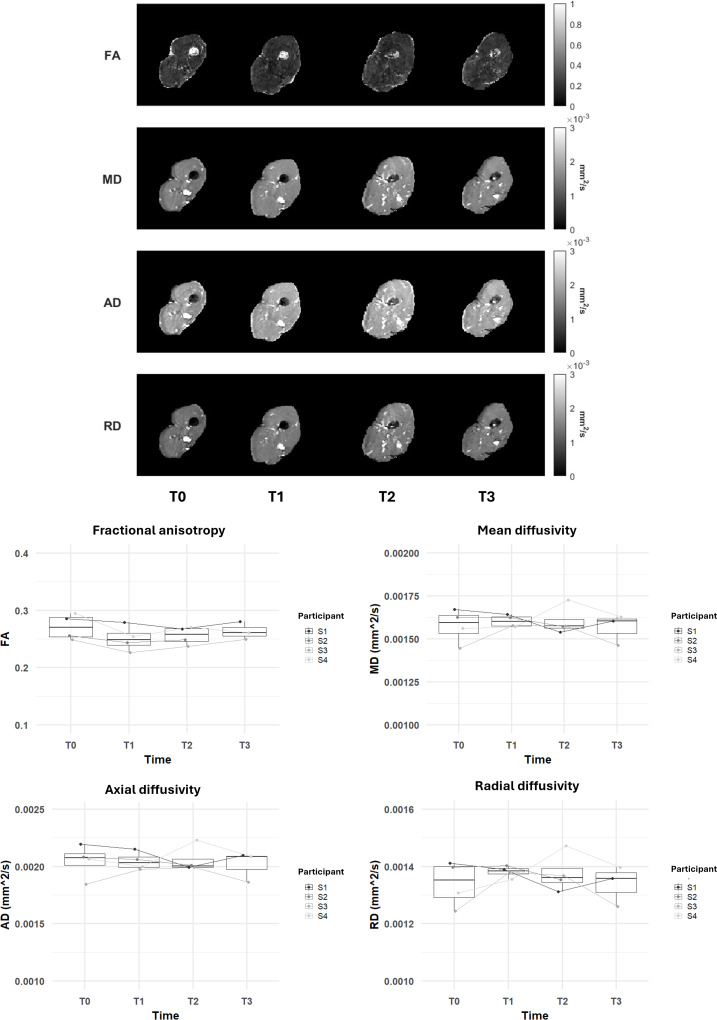
Changes in diffusion tensor imaging parameters over the 6-month functional electrical stimulation–cycling training and 1-month posttraining. The top panel displays axial magnetic resonance imaging images for fractional anisotropy, mean diffusivity, radial diffusivity, and axial diffusivity of 1 example volunteers at each time point (**T_0_, T_1_, T_2_, and T_3_**). The bottom panel shows box plots of these diffusion tensor imaging parameters for all participants over time.

As shown in Figure 8, DTI parameters revealed subtle but meaningful changes in muscle tissue microstructure.

Specifically, FA showed a decrease from 0.271 (MAD=0.03) at T_0_ to 0.249 (MAD=0.02) at T_1_, a reduction of approximately 8%. By T_2_, FA slightly increased to 0.258 (MAD=0.02), maintaining an overall reduction from baseline. At T_3_, FA increased further to 0.261 (MAD=0.017), still showing a net decrease of 4% with respect to baseline. The FA reduction was consistent in all the participants at T_1_ and T_2_.

Regarding the MD, it showed minimal variation, starting at 0.00159 mm²/s (MAD=0.00008 mm²/s) at T_0_, increasing to 0.00160 mm²/s (MAD =0.00004 mm²/s) at T_1_ and decreasing to 0.00157 mm²/s (MAD=0.00003 mm²/s) by T_2_, representing a total change of about 1%. By T_3_, MD values were at 0.00160 mm²/s (MAD=0.00003 mm²/s), showing an increase of 1% from baseline. There was no consistency among partcipants regarding any relevant trend.

As to RD, it started at 0.00135 mm²/s (MAD=0.00008 mm²/s) at T_0_, slightly increasing to 0.00138 mm²/s (MAD=0.00002 mm²/s) at T_1_, an increase of 2%. By T_2_, RD slightly decreased to 0.00136 mm²/s (MAD=0.00004 mm²/s), whereas at T_3_, RD values still persisted to 0.00136 mm²/s (MA=0.00006 mm^2^/s). At T_1_, 3 out of 4 participants exhibited the RD raise.

Finally, considering the AD, it began at 0.00207 mm²/s (MAD=0.00010 mm²/s) at T_0_, decreasing to 0.00203 mm²/s (MAD=0.00006 mm²/s) at T_1_. By T_2_, AD further decreased to 0.00200 mm²/s (MAD=0.00001 mm²/s), representing a total decrease of 4%. At T_3_, AD values were at 0.00209 mm²/s (MAD=0.00001 mm²/s), showing an increase of 1% from baseline. Even in this case, 3 out of 4 participants have shown a decrease in AD at T_1_.

## Discussion

### Principal Findings

This pilot study aimed at evaluating the feasibility of the use of mpMRI to assess the effects of 6-month FES-cycling training on muscle health in individuals with complete SCI. The use of mpMRI to evaluate SM has emerged as a novel approach recently, providing comprehensive insights into both macroscopic and microscopic changes within muscle tissues [25-27].

This advanced approach is promising for evaluating the effects of FES-cycling training on muscle tissue, moving beyond traditional metrics based essentially on the assessment of muscle volume and strength to explore changes in muscle composition and microstructural environment.

### Advanced Magnetic Resonance Imaging Insights

The relevant growth in muscle volume, and similarly the CSA increase, documented in this study highlight the capability of FES-cycling training to counteract muscle atrophy. These findings are in line with previous research that demonstrates the benefits of FES in improving muscle mass. In particular, FES-cycling training was reported to be effective in increasing CSA up to 12% in individuals with complete SCI [13-15] and more strongly electrically-stimulated resistance training, focused mainly on muscle strength and hypertrophy, have reported a 20%‐72% increase of muscle size after 8‐16 week intervention at chronic timepoints [21,22,41].

A reduction in the FF, as observed in the study in all the participants from T_0_ to T_2_, generally indicates a decrease in intramuscular fat content. This is often associated with improved muscle quality and a shift toward more lean muscle mass. In the context of FES-cycling training, this reduction likely reflects the beneficial effects of increased physical activity and muscle contractions on reducing fat infiltration in the muscles [42-44], which is commonly increased in conditions of muscle disuse or atrophy [45] such as SCI. However, previous findings on the effects of FES training on muscle fat in individuals with SCI are contrasted. Some earlier studies have failed to find any relevant change in intramuscular fat [22,46], whereas others reported up to 53% decrease [15]. It should be noted that all previous studies evaluated fat infiltration using T_1_w images in a “semiquantitative” approach, thus they did not fully exploit the capability of MRI to quantify fat infiltration using water-fat imaging techniques based on advanced Dixon techniques as proposed in our work.

Our results, although based on a limited number of participants and exhibiting significant variability, displayed a reduction in muscle water T_2_ relaxation times more consistently after 6 months of training. This reduction is typically linked to decreased muscle inflammation and fluid content, usually indicating healthier muscle conditions. More broadly, the qT_2_ of muscle water serves as an indicator of disease activity in SM [47]. Changes in qT_2_ are nonspecific and can result from various mechanisms, including inflammation, necrosis, muscular dystrophy, acute denervation, or conditions causing intracellular or extracellular edema, or a combination of both. In rehabilitation contexts, particularly following interventions like FES-cycling training, a reduction in qT_2_ may indicate positive responses to the physical activity imposed by the training regimen.

In this pilot study, we observed subtle yet meaningful changes in DTI parameters that allow us to speculate on potential modifications occurring in muscle tissue microstructure. Specifically, FA decreased from T_0_ to T_1_, and although it slightly increased at T_2_ and T_3_, it still showed a net decrease from baseline. RD increased at T_1_ and T_2_ before returning to baseline levels at T_3_. In contrast, AD decrease at T_1_ and T_2_, whereas MD showed minimal variations remaining relatively stable over the study period. These changes in DTI parameters may be related to alterations in the microstructural properties of muscle tissue following the FES-cycling training. Diffusion MRI has been widely used to assess the diffusivity of water molecules in tissue and the use of DTI was proposed to indirectly infer microstructural changes of muscle tissue [48,49]. Galbán et al [48] associated the first, second, and third eigenvalues to the diffusive transport along the long axis of a muscle fiber, within the endomysium perpendicular to the long axes of the muscle fibers, and within the cross-section of a muscle fiber, respectively. Furthermore, Hata et al [49] have associated changes in FA, AD, and RD to inflammation, regeneration and remodeling phase in a preclinical model of muscle injury. Interestingly, DTI parameters such as FA and RD have been found to be sensitive tools for monitoring muscle fiber size and can be useful in assessing muscle atrophy with some limitations in measuring muscle hypertrophy [50,51]. Furthermore, FA and RD parameters were associated with muscle fiber composition, with higher FA values (and lower RD values) indicating a higher proportion of type I fiber in muscle tissue [52]. Specifically, the decrease in FA observed in our study, primarily due to increased RD and reduced AD, may be associated with a change in muscle fiber diameters and a reconversion of fibers type, which are common responses to FES-training [53]. While it is important to acknowledge that DTI is one of the simplest methods for modeling diffusion MRI signals and that more complex modeling techniques have recently been developed for assessing SM using diffusion MRI [54-56], there remains room to speculate on interesting aspects of SM microstructure.

### Clinical and Research Implications

The findings from this study highlight the potential of mpMRI as a monitoring tool in rehabilitation settings. By providing a detailed assessment of muscle health, mpMRI can help clinicians develop more targeted and effective rehabilitation strategies. It also offers a method to supervise and adjust treatments based on individual responses, potentially leading to better outcomes and more personalized care strategies.

Furthermore, the application of mpMRI enables the exploration of the underlying mechanisms through which physical rehabilitation interventions, such as FES-cycling training, exert their effects. Understanding these mechanisms can guide the development of new interventions that target specific aspects of muscle health and function.

### Limitations and Future Directions

The limitations of this pilot study, including its small sample size and the absence of a control group, suggest caution in generalizing the findings. Future research should aim to confirm these results through larger-scale studies with diverse populations and control conditions.

The study exclusively involved male volunteers, as female participants were unavailable during the project’s s timeframe. This gender-specific enrollment, also considering the small sample size, aligns with the statistically higher occurrence of spinal cord injuries in men, which is threefold that of women, as detailed in the research by Lu et al [57]. While acknowledging this as a potential limitation, we maintain confidence in the validity of our findings. It is reasonable to expect that the observed trends in muscle parameter variations would be consistent across genders. However, it is important to note that the magnitude of changes, particularly in muscle mass, can differ in women, reflecting the distinct physiological characteristics between the sexes.

A further limitation of this study is also represented by the lack of longitudinal clinical data that, considering also the small sample size, cannot allow a reliable evaluation of relationships between MRI parameters and participant-specific characteristics (clinical, demographic etc). To strengthen the impact of the proposed approach on SM health in a rehabilitative context, larger cohort studies, including the collection of longitudinal parameters to describe patients’ characteristics, are needed.

In this study, the final timepoint, occurring one month posttraining, allowed us to evaluate the impact of deconditioning over a brief interval. This interval is representative of a potential pause in the training program, such as 1 that might occur during everyday life, for instance, a brief vacation break. Indeed, our aim was to understand the short-term reversibility of training effects and their implications for maintaining physical conditioning in real-world scenarios. It would be interesting to include an additional late MRI scan to monitor the progression of muscle atrophy and know the time required to return to baseline values. This additional data point could provide valuable insights into the recovery dynamics and inform future therapeutic strategies.

Finally, forthcoming studies should incorporate molecular and histological analyses to investigate changes at the cellular level, including muscle fiber type transitions, capillary density, and protein expression related to muscle hypertrophy and atrophy.

### Conclusions

In conclusion, this study underscores the use of mpMRI in advancing our understanding of the physiological impacts of rehabilitation interventions on muscle health in individuals with SCI. The detailed insights provided by mpMRI suggest its integration into clinical practice to assess the efficacy of interventions like FES-cycling training. By advancing our understanding of the physiological impacts of FES-cycling training, this research paves the way for more effective and personalized rehabilitation protocols, ultimately improving the quality of life for individuals with SCI.

## Supplementary material

10.2196/64825 Multimedia Appendix 1 Supplementary table

## Abbreviations

AD: axial diffusivity
CSA: cross-sectional area
CSA-max: largest cross-sectional area
DTI: diffusion tensor imaging
DWI: diffusion-weighted imaging
FA: fractional anisotropy
FES: functional electrical stimulation
FF: fat fraction
HR: heart rate
MAD: median absolute deviation
MAS: Modified Ashworth Scale
MD: mean diffusivity
mpMRI: multiparametric magnetic resonance imaging
MR: magnetic resonance
MRI: magnetic resonance imaging
Multi-TSE: multi-echo turbo spin echo
qT_2_: quantitative T_2_
RD: radial diffusivity
ROI: region of interest
SCI: spinal cord injury
SM: skeletal muscle
T_1_w: T_1_-weighted
TSE: turbo spin echo
