# Supplementary material for: Multiparametric MRI Assessment of Morpho-Functional Muscle Changes Following a 6-Month FES-Cycling Training Program: Pilot Study in People With a Complete Spinal Cord Injury
Source: JMIR Rehabil Assist Technol. 2025 Jan 16;12:e64825. doi: 10.2196/64825 (PMC11756844; doi:10.2196/64825)
Supplement: Multimedia Appendix 1 [file rehab-v12-e64825-s001.docx]

Table S1. Coefficient of Variations of MRI derived parameters.

| **Subject** | **Time-point** | **FF** | **T2** | **FA** | **MD** | **RD** | **AD** |
| --- | --- | --- | --- | --- | --- | --- | --- |
| *S1* | *T0* | 52.2 | 23.6 | 14.4 | 12.1 | 13.6 | 11.0 |
|  | *T1* | 54.5 | 17.2 | 11.7 | 8.8 | 9.7 | 8.6 |
|  | *T2* | 46.1 | 16.8 | 13.7 | 9.2 | 10.9 | 8.2 |
|  | *T3* | 44.0 | 23.7 | 13.8 | 9.9 | 11.6 | 8.8 |
|  |  |  |  |  |  |  |  |
| *S2* | *T0* | 32.5 | 12.5 | 12.9 | 7.8 | 9.5 | 6.3 |
|  | *T1* | 32.3 | 12.6 | 12.5 | 7.5 | 9.2 | 5.6 |
|  | *T2* | 35.1 | 19.1 | 13.8 | 8.5 | 11.0 | 5.6 |
|  | *T3* | 32.3 | 16.9 | 12.9 | 5.8 | 8.1 | 3.6 |
|  |  |  |  |  |  |  |  |
| *S3* | *T0* | 52.1 | 22.7 | 16.5 | 8.5 | 11.4 | 5.9 |
|  | *T1* | 42.1 | 16.6 | 14.2 | 4.3 | 6.8 | 2.7 |
|  | *T2* | 39.5 | 13.2 | 15.5 | 7.7 | 9.7 | 7.1 |
|  | *T3* | 34.1 | 19.6 | 13.5 | 6.9 | 8.8 | 5.6 |
|  |  |  |  |  |  |  |  |
| *S4* | *T0* | 31.9 | 13.8 | 15.3 | 5.4 | 6.7 | 5.5 |
|  | *T1* | 58.3 | 15.2 | 11.4 | 6.7 | 8.7 | 4.4 |
|  | *T2* | 31.4 | 7.3 | 11.9 | 4.9 | 5.6 | 5.8 |
|  | *T3* | n.a. | n.a. | n.a. | n.a. | n.a. | n.a. |

The analysis of CVs has shown that FF was the parameter with the highest variability, ranging from 31.4% to 58.3%, followed by the T2 relaxation time (7.3% to 23.7%). Regarding the DTI-derived parameters, FA showed the highest variability with CV ranging approximately from 11% to 16%, whereas all the other parameters had lower CV values ranging from 2.7% to 13.6%.

It should be noted that no relevant trends of variation could be observed in a longitudinal evaluation.
